# The Prevalence of Depression and Its Association With Quality of Life Among Pregnant and Postnatal Women in China: A Multicenter Study

**DOI:** 10.3389/fpsyt.2021.656560

**Published:** 2021-04-01

**Authors:** Hai-Xin Bo, Yuan Yang, Dong-Ying Zhang, Meng Zhang, Pei-Hong Wang, Xiao-Hua Liu, Li-Na Ge, Wen-Xuan Lin, Yang Xu, Ya-Lan Zhang, Feng-Juan Li, Xu-Juan Xu, Hong-He Wu, Todd Jackson, Gabor S. Ungvari, Teris Cheung, Yu-Tao Xiang

**Affiliations:** ^1^Department of Nursing, Peking Union Medical College Hospital, Beijing, China; ^2^Department of Public Health and Medicinal Administration, Faculty of Health Sciences, University of Macau, Macao, China; ^3^Centre for Cognitive and Brain Sciences, Institute of Advanced Studies in Humanities and Social Sciences, University of Macau, Macao, China; ^4^Department of Psychiatry, Southern Medical University Nanfang Hospital, Guangzhou, China; ^5^Department of Obstetrics, Peking Union Medical College Hospital, Beijing, China; ^6^Department of Obstetrics, Tongji Medical College, Union Medical College Hospital, Huazhong University of Science and Technology, Wuhan, China; ^7^Department of Obstetrics, Shuangliu District Maternal and Child Health Hospital, Chengdu, China; ^8^Department of Obstetrics, Shengjing Hospital, China Medical University, Shenyang, China; ^9^Department of Obstetrics, Guangdong Women and Children Hospital, Guangzhou, China; ^10^Department of Obstetrics, China-Japan Friendship Hospital, Beijing, China; ^11^Department of Obstetrics, Qinghai Provincial People's Hospital, Xining, China; ^12^Department of Nursing, Maternal and Child Health Care Hospital of Uygur Autonomous Region, Urumqi, China; ^13^Department of Obstetrics, Affiliated Hospital of Nantong University, Nantong, China; ^14^Department of Obstetrics, Nantong Maternity and Child Health Care Hospital, Nantong, China; ^15^Department of Psychology, University of Macau, Macao, China; ^16^Division of Psychiatry, School of Medicine, University of Western Australia, Perth, WA, Australia; ^17^Department of Psychiatry, University of Notre Dame, Australia, Fremantle, WA, Australia; ^18^School of Nursing, Hong Kong Polytechnic University, Hong Kong, China

**Keywords:** depression, pregnancy, postnatal period, prevalence, China

## Abstract

**Background:** This study examined the prevalence of depressive symptoms (depression hereafter) and its association with quality of life (QOL) among pregnant and postnatal women in China.

**Methods:** In this multi-center, cross-sectional study, 1,060 pregnant and postnatal women from eight hospitals were assessed. Depression and QOL were measured using the Edinburgh Postnatal Depression Scale and the World Health Organization Quality of Life Questionnaire - brief version, respectively.

**Results:** The prevalence of depression was 7.45% (95% CI: 5.87–9.04%) in the sample. Women with depression had lower QOL in physical, psychological, social and environmental domains compared to those without. Women with physical comorbidities were more likely to suffer from depression (OR = 2.391, 95% CI = 1.384–4.130, *P* = 0.002).

**Conclusion:** Due to its negative association with QOL, increased attention should be paid to depression in pregnant and postnatal women. Regular screening assessment and preventive measures should be adopted to reduce risk of depression in this population.

## Introduction

Pregnancy is characterized by multiple hormonal and anatomical changes that are associated with high risk of mental health problems such as irritability, sleep disturbances, depression and even suicidality (1, 2). Depressive symptoms (depression hereafter) during pregnancy and the postnatal period are common (3). Previous studies have found that depression is more prevalent among pregnant and postnatal women than women with physical problems such as preeclampsia, preterm childbirth and gestational diabetes (4). Depression, together with other psychological disturbances during pregnancy, are associated with a range of negative health consequences for women, their infants and families including poor infant-mother attachment, miscarriage, pre-term labor, antepartum hemorrhage, and even fetal injury (5, 6). However, depression during pregnancy and the postnatal period is often unrecognized by clinicians because it has parallels with certain pregnancy symptoms such as loss of appetite and energy, fatigue, and libido changes (7).

In order to reduce the risk of depression during pregnancy and the postnatal period and its negative impact on health outcomes, researchers should examine its epidemiology and correlates. A meta-analysis revealed that the overall prevalence of depression in pregnant and postnatal women was 11.9% (95% CI: 11.4–12.5%) (8). Another meta-analysis revealed that the overall prevalence of postpartum depression among healthy mothers was 17% (95% CI: 15–20%) (9). A range of socio-demographic factors are associated with depression during pregnancy and the postnatal phase. For instance, researchers have found that older age, lower socioeconomic status, poorer relationships with family, intimate partner violence and unplanned pregnancy are risk factors for depression among pregnant and postnatal women (4, 10–12), while comfortable living conditions, psychological resilience, and better social support perceived by women predict reduced risk for depression (13). In addition, sociocultural and economic contexts have significant associations with depression during pregnancy and following childbirth (10, 11). Therefore, its prevalence and correlates should be examined separately in different countries.

In China, some studies on depression in the perinatal period have been conducted, but findings have varied significantly, in part, because of sample differences in demographic characteristics, measurement tools, and recruitment procedures. A China-based meta-analysis revealed that the prevalence of depression during the perinatal period was 17.4% (14), but the rate decreased to 10.7% among postpartum women (15). Common limitations of previous studies conducted in China include the reliance on single study site, small sample sizes, and univariate rather than multivariate analyses as well as the failure to use specific measures on perinatal depression such as the Edinburgh Postnatal Depression Scale (EPDS) (14, 15). Instead, most previous studies used generic measures of depression such as the Patient Health Questionnaire (PHQ-9), Zung Self-Rating Depression Scale (SDS), and Beck Depression Inventory (BDI), all of which are less appropriate because normal postnatal experiences reflecting changes in appetite and sleep as well as loss of energy can be misconstrued as depressive symptoms on generic scales.

For several decades, quality of life (QOL) has been widely used as a comprehensive health outcome in clinical practice and research. For pregnant and postnatal women, the focus of QOL has expanded from preventing, detecting, and managing problems or complications to “supporting psychological adaptation to pregnancy” (16). Most past studies only examined QOL in pregnant and postnatal women with physical diseases such as gestational diabetes, and hypertension (17, 18). In contrast, little is known about the association between QOL and depression in general samples of pregnant and postnatal women. Thus, we conducted this study to examine the prevalence of depression, its demographic and clinical correlates and association with QOL among pregnant and postnatal Chinese women.

## Methods

### Study Settings and Participants

This was a multicenter, cross-sectional study conducted between February and October, 2019. Eight major hospitals in Beijing (Peking Union Medical College Hospital), Xinjiang (Maternal and Child Health Care Hospital of Uygur Autonomous Region), Liaoning (Shengjing Hospital), Guangdong (Guangdong Women and Children Hospital), Qinghai (Qinghai Provincial People's Hospital), Hubei (Huazhong University of Science and Technology Tongji Medical College), Jiangsu (Affiliated Hospital of Nantong University) and Sichuan provinces (Shuangliu District Maternal and Child Health Hospital) that are located in central, northern, southern, eastern, and western China were included to represent a range of major geographic regions in China to reduce sampling biases related to single site research and increase the sample representativeness. A consecutive patient sampling method was adopted. All women who visited Obstetrics Departments of participating hospitals for regular check-ups during daytime hours over the study period were consecutively invited to participate in this study. The inclusion criteria were as follows: (1) age 18 years or older; (2) comprehension of spoken Chinese language; (3) current pregnancy or postnatal status (i.e., from the beginning of pregnancy to 1 week after delivery); (4) provision of written informed consent. Participants were excluded if they had pre-existing psychiatric disorders such as major depressive disorder, or disturbances of consciousness that can interfere with comprehension of research measures.

### Assessment Instruments and Study Procedure

Basic demographic information including residence, maternal age, height (m), weight (kg), education, employment, family size, monthly income, pregnancy stage, status as first delivery, adverse pregnancy experience, past miscarriages and abortions, placenta preposition, physical comorbidities, were assessed on a background information form. The 10-item self-report EPDS – Chinese version (19) was used to assess severity of depressive symptoms in the past week during pregnancy or the postnatal period. Total scores ranged from 0 to 30, with higher scores indicating more severe depression. An EPDS total score of 13 or above indicates “having depression” (19). The Chinese version of EPDS had excellent psychometric properties (20). The World Health Organization Quality of Life Questionnaire – brief version (WHOQOL-BREF) was used to assess QOL. The WHOQOL-BREF consists of 26 items, covering physical, psychological, environmental and social QOL (21). A higher total score indicates better QOL (22). The Chinese version of this scale has good psychometric properties (23).

Following other studies (24, 25), three standardized “Yes/No” questions were utilized to evaluate suicide risk factors in the past year, including (1) suicidal ideation – “Have you ever thought about killing yourself?,” (2) Suicide plans – “Have you ever made a plan for committing suicide, or even taken steps to prepare for this plan?,” and (3) Suicide attempts – “Have you ever attempted suicide?.” Participants who answered “Yes” to any of the three questions were considered as “having suicidality.”

All participants were approached by trained nurses who explained the study aim and procedure. For those who agreed to participate and provided written informed consent form, a face-to-face interview was conducted. This study was approved by the Clinical Research Ethics Committee of the Peking Union Medical College Hospital and all participating hospitals (Ref No: S-K1273). All the study procedures were carried out in accordance with relevant guidelines.

### Sample Size Estimation

The total sample size of participants was estimated using a standardized formula: *N* = $Z_{\alpha }^{2}$ *P* (1 – *P*)/*d*^2^ (26). *N* means the sample size, *Z* means the statistic of significance test, alpha means the significance level, *P* means the prevalence, and *d* means the allowable error. In this study, alpha was set at 0.05, *Z*_α_ = 1.96, and the estimated acceptable margin of effort for proportion *d* was 0.1 as recommended (26). The prevalence of depression in pregnant and postnatal women was 15% in a systematic review (27), which was used for sample size calculation in this study. A minimum sample size of 490 participants were needed. To increase the statistical power, we recruited 1,100 participants in this study.

### Statistical Analyses

Data analyses were performed using SPSS V24.0. Normality distributions of continuous variables were checked by one-sample Kolmogorov-Smirnov tests. Differences on demographic and clinical characteristics between depression and no depression groups were assessed using independent samples *t*-tests for normally distributed continuous variables, Mann-Whitney U tests for non-normally distributed continuous variables, and Chi-square tests/Fisher exact tests for categorical variables. Analysis of covariance (ANCOVA) was used to compare QOL between depression and no depression groups after controlling for variables on which these groups differed (*P* < 0.05) in univariate analyses. Binary logistic regression analysis based on the “enter” method was used to examine independent correlates of depression. Depression was the dependent variable, while variables on which there were significant univariate group differences were entered as independent variables. Significance was set at *P* < 0.05, with two-tailed tests.

## Results

A total of 1,140 women were invited to participate in this study; of these, 1,060 agreed and completed the assessment, producing a response rate of 92.98%. The overall prevalence of depression was 7.45% (95% CI: 5.87–9.04%); within the depressed subgroup (*N* = 79), the highest rate was found in the third trimester (45.6%), followed by the first trimester (25.3%) and second trimester (19.0%). The lowest figure was shown among postnatal women (10.1%). Within the entire sample, 27 (2.6%) women reported having experiences of suicidality, with 26 (2.5%) reporting suicidal ideation, 4 (0.38%) reporting a suicide plan and 5 (0.47%) admitting to a suicide attempt. Specifically, there were 14 women (17.7%) with suicidality in the depression group compared to 13 women (1.3%) in the no depression group (*P* < 0.001). Demographic and clinical characteristics are presented in Table 1. EPDS ratings are presented in Table 2.

**Table 1:** Demographic characteristics of the Chinese pregnant women (*N* = 1,060).

							**Univariate analyses**			**Multivariate logistic regression**		
**Variable**	**Total (*****N*** **=** **1,060)**		**No depression (*****N*** **=** **981)**		**Depression (*****N*** **=** **79)**		**X^**2**^/Z**	**df**	***P***	**OR**	**95% CI**	***P***
	***N***	**%**	***N***	**%**	***N***	**%**						
Urban residence	897	84.6	834	85.0	63	79.7	1.560	1	0.212	–	–	–
**Pregnancy**												
First trimester	200	18.9	180	18.3	20	25.3	2.443	3	0.486	–	–	–
Second trimester	235	22.2	220	22.4	15	19.0						
Third trimester	516	48.7	480	48.9	36	45.6						
Postnatal	109	10.3	101	10.3	8	10.1						
College and above	712	67.2	665	67.8	47	59.5	2.281	1	0.131	–	–	–
Employed	655	61.8	608	62.0	47	59.5	0.191	1	0.662	–	–	–
Having four and more family members	526	49.6	487	49.6	39	49.4	0.002	1	0.962	–	–	–
Monthly income ≥ 5,000 RMB	509	48.0	478	48.7	31	39.2	2.635	1	0.105	–	–	–
First delivery	608	57.4	563	57.4	45	57.0	0.005	1	0.941	–	–	–
Adverse event during pregnancy	140	13.2	124	12.6	16	20.3	3.697	1	0.055	–	–	–
Previous natural miscarriage	203	19.2	181	18.5	22	27.8	4.170	1	**0.041**	1.407	0.795–2.491	0.242^b^
Previous drug-induced abortion	312	29.4	280	28.5	32	40.5	5.038	1	**0.025**	1.485	0.885–2.494	0.135^c^
Placenta preposition	62	5.8	55	5.6	7	8.9	–^a^	–	0.216	–	–	–
Physical comorbidity	139	13.1	119	12.1	20	25.3	11.157	1	**0.001**	2.391	1.384–4.130	**0.002**^d^
							**Univariate analyses**			**ANCOVA**		
	**Mean**	**SD**	**Mean**	**SD**	**Mean**	**SD**	***T***	**df**	***P***	**F**	**df**	***P***
Age (years)	29.41	4.21	29.41	4.19	29.44	4.57	−0.052	1058	0.959	–	–	–
BMI	24.10	4.24	24.13	4.29	23.82	3.68	0.613	1058	0.540	–	–	–
Physical QOL	15.21	2.07	15.35	2.02	13.45	1.91	8.108	1058	** <0.001**	60.874	1	** <0.001**^e^
Psychological QOL	15.26	2.44	15.46	2.34	12.80	2.21	9.733	1058	** <0.001**	91.107	1	** <0.001**^e^
Social QOL	15.57	2.37	15.72	2.30	13.61	2.37	7.815	1058	** <0.001**	57.736	1	** <0.001**^e^
Environmental QOL	15.04	2.49	15.24	2.39	12.66	2.53	9.185	1058	** <0.001**	81.396	1	** <0.001**^e^
EPDS Total score	5.43	4.40	4.64	3.44	15.30	2.64	–	–	–	–	–	–

*Marital status was not included because all participating women reported a current marital relationship*.

a *Fisher's Exact Test*.

b *Using age, abortion by drugs, and comorbidity as covariates*.

c *Using age, natural miscarriage, and comorbidity as covariates*.

d *Using age, natural miscarriage, and abortion by drugs as covariates; e: using natural miscarriage, abortion by drugs, and comorbidity as covariates*.

*BMI, Body mass index; CI, Confident Interval; EPDS, Edinburgh Postnatal Depression Scale; QOL, Quality of life. Bold value indicates P < 0.05*.

**Table 2:** EPDS ratings.

	**Mean**	**SD**	***N*** **(%)**			
**Items**			**Score = 0**	**Score = 1**	**Score = 2**	**Score = 3**
EPDS-1	0.225	0.497	847 (79.9)	157 (14.8)	37 (3.5)	1 (0.1)
EPDS-2	0.203	0.471	866 (81.7)	149 (14.1)	30 (2.8)	1 (0.1)
EPDS-3	0.906	0.784	366 (34.5)	419 (39.5)	242 (22.8)	13 (1.2)
EPDS-4	0.783	0.717	383 (36.1)	533 (50.3)	102 (9.6)	27 (2.5)
EPDS-5	0.488	0.689	641 (60.5)	303 (28.6)	83 (7.8)	13 (1.2)
EPDS-6	0.392	0.584	689 (65.0)	300 (28.3)	53 (5.0)	1 (0.1)
EPDS-7	0.867	0.799	399 (37.6)	397 (37.5)	227 (21.4)	17 (1.6)
EPDS-8	0.551	0.699	594 (56.0)	322 (30.4)	121 (11.4)	3 (0.3)
EPDS-9	0.897	0.814	393 (37.1)	383 (36.1)	251 (23.7)	17 (1.6)
EPDS-10	0.120	0.384	937 (88.4)	82 (7.7)	20 (1.9)	1 (0.1)
EPDS total	5.4315	4.396	–	–	–	–

*EPDS, Edinburgh Postnatal Depression Scale*.

In univariate analyses, depression was significantly associated with previous natural miscarriage experiences (*P* = 0.041), drug-induced abortion history (*P* = 0.025) and physical comorbidities (*P* = 0.001) which were controlled for as covariates in subsequent analyses. Women with depression had lower physical, psychological, social and environmental quality of life compared to those without (all *P*-values < 0.001) after controlling for covariates. A multivariate logistic regression analysis revealed that women with physical comorbidities were more likely to suffer from depression (OR = 2.391, 95% CI = 1.384–4.130, *P* = 0.002) (Table 1).

## Discussion

Due to hormonal and anatomical changes and other factors including heavier care burdens, pregnant and postnatal women are more likely to suffer from psychological disturbances, particularly depression (28). In this study, we found that the prevalence of depression was 7.45% (95% CI: 5.87–9.04%) among pregnant and postpartum women. In addition, women with depression had lower QOL in physical, psychological, social and environmental domains compared to those without. Women with physical comorbidities were more likely to suffer from depression.

The prevalence of depression in this study was lower than estimates from recent China-based meta-analyses (14, 15). Notably, however, rates have varied between reviews. For example, an older meta-analysis (29) found that the pooled point prevalence of perinatal depression ranged from 6.5 to 12.9% from the start of pregnancy to the first postpartum year; 19.2% of the depressed subgroup reported depression during the first 3 months after delivery. Conversely, another recent meta-analysis found the overall prevalence of depression in pregnant and postnatal women was 11.9% (95% CI: 11.4–12.5%) (8). Discrepant rates between studies could be partly explained by different study samples, sampling methods, sample sizes, measurement tools for depression, socioeconomic backgrounds and clinical status (8, 9, 29).

Previous studies have indicated that depression is a strong predictor of suicidality (30–32). In this study, a relatively small proportion of women reported suicidal ideation (2.5%), plan (0.38%) or attempt (0.47%) but the rate was substantially higher than those of other studies. For example, a large retrospective study from the US found that the prevalence of suicide attempts during pregnancy was 0.04% (33). Another study from Canada reported that the prevalence of suicide attempts was 0.03% in pregnant women and 0.06% in postnatal women (34). A study conducted in mainland China found that the prevalence of suicide attempt during pregnancy was 0.21% (35). Reasons for the comparatively higher rate in this research are not known. However, due to differences in timeframes and measures of suicidality between studies, direct comparisons should be made with caution. In addition, we found that pregnant and postnatal women with depression were more likely to have suicidality compared with those without, which is consistent with previous findings (1, 36).

Additionally, our study found that participants with physical comorbidities were more likely to report depression, which also dovetails with previous findings (37, 38). Physical comorbidities and adverse effects of treatments in pregnant and postnatal women are associated with more severe physical discomfort and impaired daily functioning, which could increase the risk of depression (37, 38).

Similar to previous findings (39), depression was significantly associated with lower QOL in all domains. The poorer QOL of depressed women could be explained by the distress/protection QOL model, in which QOL is determined by a range of protective and distressing factors (40). QOL tends to be lower if distressing factors (e.g., frequent sleep disturbances, fatigue, and physical discomfort caused by depression) predominate overprotective factors (e.g., better social support from social networks). Depressed women often present with psychological and physical symptoms such as sadness, helplessness, cognitive impairments, body pain, insomnia, and digestive problems (41), all of which are related to lower QOL.

The merits of this study included its multicenter study design, large sample size, and use of a depression measure validated specifically for pregnant and postnatal depression. The main limitations should also be acknowledged. First, this was a cross-sectional study, so causal associations and changes between variables over time could not be examined. Second, all results were based on self-reported data; therefore, we cannot rule out biases in recall or social desirability as influences on the data. Third, while the QOL scale provided a comprehensive assessment of the women's perceptions of their physical health, psychological well-being, social relationships, and physical environments, number of dependent children and extended family members in the household could have been assessed with more specificity than the included item (presence of four or more family members in the household). Fourth, although a relatively large sample size and multicenter design based on different geographic regions of China were used, the needed number of eligible perinatal women was not calculated separately for each participating hospital. Finally, assessments were limited to women who visited hospital Obstetrics Departments for regular check-ups during daytime hours; we viewed this strategy as ethical but also acknowledge it is related to potential selection biases. For example, results may not apply to women who visited emergency departments during non-daytime hours. Such groups were not included for reasons related to both logistics and clinical care (i.e., limited staff during non-daytime hours, the priority of attending to acute concerns of emergency room attendees rather than having them complete a research study during a time of crisis). In addition, results may not generalize to women with pre-existing psychiatric disorders and potential comprehension difficulties were excluded. To increase population representativeness, emergency room contacts might be assessed following the resolution of immediate crises, and more costly national epidemiological surveys using multistage designs, random sampling, and additional research staff require funding support in China and other nations.

## Conclusion

In conclusion, due to the significant associations of depression with suicidality, and lower QOL, increased attention should be paid to pregnant and postnatal women. Preventive measures, such as regular assessment and management of physical comorbidities, should be adopted to track and reduce risk of depression in this population. Timely treatment should be provided for women who experience depression during pregnancy and the postnatal period.

## Data Availability Statement

The Clinical Research Ethics Committee of Peking Union Medical College Hospital that approved the study prohibits the authors from making the research data set publicly available. Readers and all interested researchers may contact Dr. Hai-Xin Bo (Email address: bohxin@126.com) for details. Dr. Hai-Xin Bo could apply to the Clinical Research Ethics Committee of Peking Union Medical College Hospital for the release of the data.

## Ethics Statement

The studies involving human participants were reviewed and approved by Clinical Research Ethics Committee of the Peking Union Medical College Hospital and all participating hospitals (Ref No.: S-K1273). The patients/participants provided their written informed consent to participate in this study.

## Author Contributions

H-XB, YY, and Y-TX: study design. YY, D-YZ, MZ, P-HW, X-HL, L-NG, W-XL, YX, Y-LZ, F-JL, X-JX, and H-HW: data collection, analysis and interpretation. YY, TC, and Y-TX: drafting of the manuscript. TJ and GU: critical revision of the manuscript. All authors: approval of the final version for publication.

## Conflict of Interest

The authors declare that the research was conducted in the absence of any commercial or financial relationships that could be construed as a potential conflict of interest.
